# Limitations of the nested reverse transcriptase polymerase chain reaction on tyrosinase for the detection of malignant melanoma micrometastases in lymph nodes

**DOI:** 10.1054/bjoc.2000.1282

**Published:** 2000-06-15

**Authors:** A Calogero, H Timmer-Bosscha, H Schraffordt Koops, A T M G Tiebosch, N H Mulder, G A P Hospers

**Affiliations:** Departments of Medical Oncology, Surgical Oncology, Pathology and Laboratory Medicine, University Hospital Groningen, PO Box 30.001

**Keywords:** tyrosinase, melanoma, lymph nodes, nested RT-PCR

## Abstract

The specificity and sensitivity of the nested reverse transcriptase polymerase chain reaction (RT-PCR) on tyrosinase was studied, for the detection of micrometastases of malignant melanoma. The specificity was assessed in the blood of six healthy donors, four patients with non-melanoma cancers of which one patient was treated with granulocyte-colony stimulating factor. Lymph nodes of nine patients without malignant melanoma were tested and four cell lines of various other tumours. Six of the nine non-melanoma lymph nodes were positive in this assay. The sensitivity was tested in a spike experiment in vitro, using a melanoma cell line. The detection limit was ten melanoma cells per 10^7^peripheral blood lymphocytes. © 2000 Cancer Research Campaign


*British Journal of Cancer* (2000) **83**(2), 184–187
© 2000 Cancer Research Campaign
doi: 10.1054/ bjoc.2000.1282, available online at http://www.idealibrary.com on IDEAL®

### Limitations of the nested reverse transcriptase polymerase chain reaction on tyrosinase for the detection of malignant melanoma micrometastases in lymph nodes

**A Calogero[1], H Timmer-Bosscha[1], H Schraffordt Koops[2], ATMG Tiebosch[3], NH Mulder[1] and GAP Hospers[1]**

Departments of [1]Medical Oncology, [2]Surgical Oncology, and [3]Pathology and Laboratory Medicine, University Hospital Groningen, PO Box 30.001, 9700 RB Groningen, The Netherlands

**Summary** The specificity and sensitivity of the nested reverse transcriptase polymerase chain reaction (RT-PCR) on tyrosinase was studied, for the detection of micrometastases of malignant melanoma. The specificity was assessed in the blood of six healthy donors, four patients with non-melanoma cancers of which one patient was treated with granulocyte-colony stimulating factor. Lymph nodes of nine patients without malignant melanoma were tested and four cell lines of various other tumours. Six of the nine non-melanoma lymph nodes were positive in this assay. The sensitivity was tested in a spike experiment in vitro, using a melanoma cell line. The detection limit was ten melanoma cells per $10^7$ peripheral blood lymphocytes. © 2000 Cancer Research Campaign

**Keywords:** tyrosinase; melanoma; lymph nodes; nested RT-PCR

Treatment for localized malignant melanoma is difficult and the prognosis is still uncertain in many cases. Although clinical parameters such as lymph node involvement and thickness of the tumour are helpful, other diagnostic tools are urgently needed to allow for optimal prediction of the clinical course and choice of treatment. For this purpose efforts have been made to detect micrometastases in blood (Brossart et al, 1995; Foss et al, 1995; Hoon et al, 1995; Buzaid and Balch 1996; Mellado et al, 1996; Gläser et al, 1997; Jung et al, 1997; Reinhold et al, 1997; Farthmann et al, 1998; Ghossein et al, 1998; Curry et al, 1998; De Vries et al, 1999; Palmieri et al, 1999) and in the regional lymph nodes by immunohistochemistry or reverse transcriptase polymerase chain reaction (RT-PCR) (Battyani et al, 1993; Wang et al, 1994; Rankin, 1996; Van der Velde-Zimmermann et al, 1996; Blaheta et al, 1998; Goydos et al, 1998; Hatta et al, 1998; Bieligk et al, 1999).

Among these techniques the nested RT-PCR is the most sensitive method: in spike experiments the detection limit ranges from 1 to 10 tumour cells in $10^7$ peripheral blood lymphocytes (PBLs) (Hoon et al, 1995; Gläser et al, 1997; Jung et al, 1997; Farthmann et al, 1998; Palmieri et al, 1999), whereas for immunohistochemistry the detection limit is 1 tumour cell in $10^5$ normal cells (Hatta et al, 1998).

The expression of the tyrosinase gene is most widely used for the detection of circulating melanoma cells due to its high percentage of expression in malignant melanoma.

In 1991, Smith et al reported that circulating melanoma cells can be detected by amplification of tyrosinase mRNA. The initial report led to a number of more detailed investigations regarding the presence of tyrosinase RNA in peripheral blood of melanoma patients (Brossart et al, 1995; Foss et al, 1995; Hoon et al, 1995; Buzaid and Balch, 1996; Mellado et al, 1996; Gläser et al, 1997; Jung et al, 1997; Reinhold et al, 1997; Curry et al, 1998; Farthmann et al, 1998; Ghossein et al. 1998; Palmieri et al, 1999; De Vries et al, 1999). The published tyrosinase mRNA RT-PCR sensitivities in peripheral blood among stage III melanoma patients range from 0% to 100%. Based on these results and their own findings in 102 melanoma patients, Gläser et al (1997) concluded that the detection of circulating tumour cells in melanoma patients using the tyrosinase mRNA RT-PCR is not sensitive enough for early detection.

Non-melanoma blood controls were, however, always negative for expression of tyrosinase, using the nested RT-PCR; nevertheless, whether the same specificity can be reached for lymph nodes is doubtful for two reasons. First, although evidently other tyrosinase positive cells than melanoma cells, such as Schwann cells or melanocytes, do not circulate, they could well be present in the stroma of normal nodes. Secondly, studies of lymph nodes of patients with stage I or II malignant melanoma (AJCC classification), thus with clinically negative nodes, showed results that do not seem to correspond with the clinical outcome. For instance Wang et al (1994) found in 66% of 29 regional lymph node samples tyrosinase positivity by RT-PCR, in melanoma patients with a stage I or II melanoma. Therefore, the specificity of the tyrosinase mRNA as a tumour marker in lymph nodes should be further evaluated.

In the present study we tried to evaluate both sensitivity and specificity for the detection of tyrosinase mRNA by means of nested RT-PCR, both in blood and in lymph nodes.

*Received 13 August 1999*
*Revised 31 January 2000*
*Accepted 7 March 2000*

*Correspondence to:* GAP Hospers

### MATERIALS AND METHODS

### Patients

For the use of human material informed consent and medical ethical approval was obtained according to local hospital rules.

Blood was taken from six healthy donors and four patients with a malignancy other than melanoma, of which one patient with breast cancer was treated with gramulocyte colony-stimulating factor (G-CSF).

Nine lymph nodes of patients without malignant melanoma were analysed; by conventional haematoxylin and eosin (H&E) staining in combination with immunohistochemistry only reactivity was found in these lymph nodes. Among these patients, three did have malignancy: one patient had a leiomyosarcoma grade II of the uterus, one patient a non-seminoma testis stage I, and one a cholangiocarcinoma. In the other six patients no malignancy was found. Among these patients, two received immunosuppressive therapy, following a renal and a liver transplantation; in the other four only a reactive lymph node was found.

### Surgical specimens

Lymph nodes were obtained by standard surgical procedures. All tissues were collected and dissected under stringent sterile conditions to prevent RNA contamination. In the processing of the samples special care was taken to prevent contact with skin in order to avoid contamination with skin melanocytes.

Each lymph node was bisected: 4-μm frozen sections were obtained from each half of the lymph node. These were immediately stored at –80°C until use for the nested RT-PCR. The remaining tissue was formalin-fixed and paraffin-embedded and, using standard procedures, examined both by conventional H&E staining and by immunohistochemistry with S-100, HMB45 and MAT-1 antibodies. By the latter analysis no tumour cells could be detected.

### Cell lines

The melanoma cell line SK23-mel was a gift of P Schrier (University Hospital of Leiden, The Netherlands). Non-melanoma cell lines used were: A2780, ovarian cancer (Rogan et al, 1984), GLC16, lung cancer (Berendsen et al, 1988), NT2/D1, testis cancer (Andrews et al, 1984), SW948, colon cancer (Leibovitz et al, 1976). All the cell lines were grown under standard cell culture conditions.

### Primers

All primers were synthesized by Eurogentech (Searing, Belgium). The sequences are:

HTYR1=TTGGCAGATTGTCTGTAGCC (outer, sense)
HTYR2=AGGCATTGTGCATGCTGCTT (outer, anti-sense)
HTYR3=GTCTTTATGCAATGGAACGC (nested, sense)
HTYR4=GCTATCCCAGTAAGTGGACT (nested, anti-sense)
β-actin sense=ACCACACCTTCTACAATGAGCTGCGTG
β-actin anti-sense=CACAGCTTCTCCTTAATGTCACGCACG.

### Antibodies

The anti-tyrosinase antibody MAT-1 was purchased from Brunschwig Chemie BV (Amsterdam, The Netherlands). The antibody S-100 was purchased from Dako (Glostrup, Denmark).

### Reagents

All enzymes and reagents used in this study were purchased from Gibco-BRL (Breda, The Netherlands).

### Immunhistochemistry

Briefly, acetone fixed slides were stained by a standard two-step immunoperoxidase staining protocol; the second step was performed using a rabbit anti-mouse horseradish peroxidase-labelled antibody (Dako, Glostrup, Denmark).

### PBL isolation

For peripheral blood samples, the first vacuum tube of blood drawn after venipuncture was discarded. For each sample, 16 ml of peripheral venous blood were collected in EDTA containing tubes. The blood was stored at 4°C and processed within 2–4 h. Erythrocytes were lysed by incubating the samples with 30–40 ml erythrocyte lysis buffer (155 mM ammonium chloride, 10 mM potassium hydrogen carbonate, 0.1 mM potassium EDTA) in ice for 10 min twice. After centrifugation pellets were washed twice in phosphate-buffered saline solution (PBS) and stored at –20°C in RNA extraction buffer GIT (4 M guanidiumthiocynate, 2% β-mercaptoethanol).

### RNA isolation

For all samples the total RNA was extracted with a kit (Qiagen, Westburg BV, Leusden, The Netherlands), according to the manufacturer’s prescriptions. The amount and integrity of the extracted RNA was evaluated by agarose gel electrophoresis.

### c-DNA synthesis

Single-stranded c-DNA synthesis was carried out on 1 μg total RNA with 25 pmole of each anti-sense β-actin and the tyrosinase-specific HTYR2 primers together, according to the manufacturer’s instructions. To prevent contamination, c-DNA synthesis, PCR mix preparation and agarose electrophoresis were performed in separate rooms.

### Nested RT-PCR

For the first PCR reaction 1/10 of the c-DNA synthesis mix was used. The reaction was carried out in 25 μl with 100 pmole of tyrosinase primers (HTYR1, HTYR2) and β-actin primers together, 1.5 mM megnesium chloride and 200 μM of each dNTP. The reaction was carried out for 30 cycles, each cycle consisting of: 1 min 95°C; 1 min 55°C; 1 min 72°C. Then 10 μl of each sample were loaded on a 2% agarose gel. The presence of the correct β-actin band (365 bp) indicates both the quality of the RNA and the success of the first PCR reaction. The nested PCR was performed using as a template a 1 to 100 dilution of the product of the first PCR, with the same mix and cycle condition as for the first PCR, but using primers HTYR3 and HTYR4. The reaction were performed in a DNA thermal cycler (Perkin-Elmer, Applied Biosystems, Nieuwekerk a/d IJssel, The Netherlands).

*© 2000 Cancer Research Campaign*

### Spike experiment

An homogenous suspension of SK23-mel cells was serially diluted to a concentration ranging from 1 to $10^4$ cells 100 $\mu l^{-1}$ PBS. Subsequently each cells suspension was added to 10 ml of blood and the mixture was normally processed for PBLs isolation, c-DNA synthesis and nested RT-PCR, as described above.

### RESULTS

### Lymph nodes

Tyrosinase was detected by nested RT-PCR in 6/9 lymph nodes of non-melanoma patients (Table 1, Figure 1). By immunohistochemistry none of the LNs showed aberrant cells with S-100 expression apart from the normal expression in interdigitating cells, for either expression of tyrosinase using the MAT-1 antibody could not be detected.

### Cell lines

Tyrosinase was found to be positive by nested RT-PCR in 2/4 of the tested cell lines: colon cell line SW948 and ovarian cell line A2780; on the other hand none of the cell line tested was positive for immunocytochemistry, with the anti-tyrosinase antibody MAT-1.

### Blood samples

All non-melanoma patients, including the patient with breast cancer treated with G-CSF (*n* = 4) and all healthy donors (*n* = 6) were negative for the nested RT-PCR on tyrosinase in the blood, suggesting a reasonable degree of specificity (0/10) for this assay in blood. The blood sample of the patient treated with G-CSF permitted the analysis of any effect of young leukocytes on the specificity of the assay.

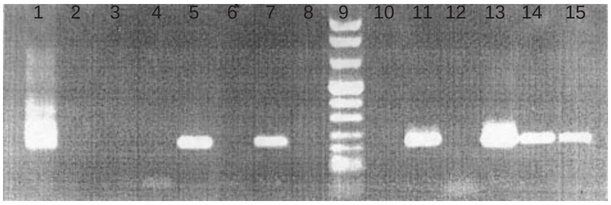

**Figure 1** A 2% agarose gel with the product of the nested RT-PCR on lymph nodes, as a 207 bp band. For diagnosis of patient no. see Table 1. Slots: 1. Positive control: melanoma tissue; 2. Negative control: blood from a healthy donor; 3. Negative control: blood from a healthy donor; 4. Negative control: no template; 5. Patient no. 1; 6. Negative control: no template; 7. Patient no. 2; 8. Patient no. 3; 9. Marker: 1114 bp, 900 bp, 692 bp, 501–489 bp, 404 bp, 320 bp, 242 bp, 190 bp, 147 bp, 124 bp, 110 bp, 67 bp, 37–34–26–19 bp; 10. Patient no. 4; 11. Patient no. 5; 12. Patient no. 6; 13. Patient no. 7; 14. Patient no. 8; 15. Patient no. 9.

**Table 1** Results obtained on non-melanoma patients' lymph nodes, with the nested RT-PCR assay

| Patients' diagnosis[a] | Results with nested RT-PCR |
|---|---|
| no-malignancy (*n* = 4); Patient nos 1, 2, 3, 4 | 50% positive |
| Leiomyosarcoma (*n* = 1); Patient no. 5 | Positive |
| Non-seminoma testis (*n* = 1); Patient no. 6 | Negative |
| Cholangiocarcinoma (*n* = 1); Patient no. 7 | Positive |
| Liver transplanstation (*n* = 1); Patient no. 8 | Positive |
| Renal transplanstation (*n* = 1); Patient no. 9 | Positive |

[a]For agarose gel and no. see Figure 1.

### Spike experiment

To determine the sensitivity of the nested RT-PCR assay we performed a spike experiment as described in Materials and Methods. The results indicated a detection limit of 10 SK23-mel cells in $10^7$ PBLs.

### DISCUSSION

The aim of this study was to determine specificity and sensitivity of the nested RT-PCR on tyrosinase for the detection of micrometastases in lymph nodes of malignant melanoma patients.

We first checked a number of lymph node biopsies of non-melanoma patients. We found 6/9 positive samples. Contamination of skin cells during the sample processing can not be excluded completely, although this was done under very stringent conditions.

These results are conflicting with the recent study by Blaheta et al (1998) who found no positive results in lymph nodes of 40 non-melanoma patients. However, it is to be noted that in this study, together with the nested RT-PCR an immunohistochemical assay, is performed and eventual positive results are judged to be positive only when confirmed by the detection of tyrosinase-positive cells by means of immunohistochemistry. Thus, specificity in this study is determined by immunohistology, which is ten times less sensitive than nested RT-PCR.

On the other hand, considering the specificity of the nested RT-PCR, our finding of positive results in lymph nodes of non-melanoma patients is in agreement with the publications of Bieligk et al (1998) and of Battyani et al (1993), who found tyrosinase transcripts in a variety of normal organs, including lymph nodes, but not in blood.

These results may be due to the presence of other tyrosinase-positive cells than melanoma cells, like Schwann cells or melanocytes. The presence of Schwann cells or melanocytes in LNs is a commonly described phenomenon, and it has been reported to range from 7.3% to 22% (Bautista et al, 1994).

A second cause for tyrosinase expression in lymph nodes could be the phenomenon called 'illegitimate transcription' (Chelly et al, 1989), by which, due to a 'leaky' transcription regulation, some 'luxury' genes are expressed also in 'contexts' where they are supposed to be off.

This phenomenon is probably the explanation for the positive PCR results we found in 2/4 non-melanoma cell lines.

In conclusion, the nested RT-PCR on tyrosinase to detect the presence of malignant melanoma micrometastases in lymph nodes has limited diagnostic value, due to the high percentage of false-positive results in lymph nodes of non-melanoma patients.

 *© 2000 Cancer Research Campaign*

### REFERENCES

Andrews PW, Damjanov I, Simon D, Banting GS, Carlin C, Dracopoli NC and Fogh J (1984) Pluripotent embryonal carcinoma clones derived from the human teratocarcinoma cell line tera-2. Differentiation in vivo and in vitro. *J Lab Invest* **50**: 147–162

Battyani Z, Xerri L, Hassoun J, Bonerandi JJ and Grob JJ (1993) Tyrosinase gene expression in human tissues. *Pigment Cell Res* **6**: 400–405

Bautista NC, Cohen S and Anders KH (1994) Benign melanocytic nevus cells in axillary lymph nodes: a prospective incidence and immunohistochemical study with literature review. *Am J Clin Pathol* **102**: 102–108

Berendsen HH, De Ley L, De Vries EGE, Mesander G, Mulder NH, De Jong B, Buys CHCM, Postmus PE, Poppema S, Sluiter HJ and The HT (1988) Characterization of three small cell lung cancer cell lines established from one patient during longitudinal follow-up. *Cancer Res* **48**: 6891–6899

Bieligk SC, Ghossein R, Bhattacharya S and Coit DG (1999) Detection of tyrosinase mRNA by reverse transcription-polymerase chain reaction in melanoma sentinel nodes. *Ann Sur Oncol* **6**: 232–240

Blaheta H-J, Schittek B, Breuninger H, Maczey E, Kroeber S, Sotlar K, Ellwanger U, Thelen MH, Rassner G, Bultmann B and Grabe C (1998) Lymph node micrometastases of cutaneous melanoma: increased sensitivity of molecular diagnosis in comparison to immunohistochemistry. *Int J Cancer* **79**: 318–323

Brossart P, Schmier JW, Kruger S, Willhauck M, Scheibenbogen C, Moler T and Keilholz U (1995) A polymerase chain reaction-based semiquantitative assessment of malignant melanoma cells in peripheral blood. *Cancer Res* **55**: 4065–4068

Buzaid AC and Balch CM (1996) Polymerase chain reaction for detection of melanoma in peripheral blood: too early to assess clinical value. *J Natl Cancer Inst* **88**: 590–594

Chelly J, Concordet JP, Kaplan JC, and Khan A (1989) Illegitimate transcription: transcription of any gene in any cell type. *Proc Natl Acad Sci USA* **86**: 2617–2621

Curry BJ, Myers K and Hersey P (1998) Polymerase chain reaction detection of melanoma cells in the circulation: relation to clinical stage, surgical treatment, and recurrence from melanoma. *J Clin Oncol* **16**: 1760–1769

De Vries TJ, Fourkour A, Punt CJA, Van de Locht LTF, Wobbes T, Van den Bosch S, De Rooij MJM, Mensink EJBM, Ruiter DJ and Van Muijen GNP (1999) Reproducibility of detection of tyrosinase and MART-1 transcripts in the peripheral blood of melanoma patients: a quality control study using real-time quantitative RT-PCR. *Br J Cancer* **80**: 883–891

Farthmann B, Eberle J, Krasagakis K, Gstottner M, Wang N, Bisson S and Orfanos CE (1998) RT-PCR for tyrosinase-mRNA-positive cells in peripheral blood: evaluation strategy and correlation with known prognostic markers in 123 melanoma patients. *J Invest Dermatol* **110**: 263–267

Foss AJE, Guille MJ, Occleston NL, Hykin PG, Hungerford JL and Lightman S (1995) The detection of melanoma cells in peripheral blood by reverse transcription-polymerase chain reaction. *Br J Cancer* **72**: 155–159

Ghossein RA, Coit D, Brennan M, Zhan ZF, Wang Y, Bhattacharya S, Houghton A and Rosai J (1998) Prognostic significance of peripheral blood and bone marrow tyrosinase messenger RNA in malignant melanoma. *Clin Cancer Res* **4**: 419–428

Gläser R, Rass K, Seiter S, Hauschild A, Christopers E and Tilgen W (1997) Detection of circulating melanoma cells by specific amplification of tyrosinase complementary DNA is not a reliable tumour marker in melanoma patients: a clinical two-centre study. *J Clin Oncol* **15**: 2818–2825

Goydos JS, Ravikumar TS, Germino FJ, Yudd A and Bancila E (1998) Minimally invasive staging of patients with melanoma: sentinel lymphoadenectomy and detection of the melanoma-specific proteins MART-1 and tyrosinase by reverse transcriptase polymerase chain reaction. *J Am Coll Surg* **187**: 182–190

Hatta N, Takata M, Takehara K and Ohara K (1998) Polymerase chain reaction and immunohistochemistry frequently detect occult melanoma cells in regional lymph nodes of melanoma patients. *J Clin Pathol* **51**: 597–601

Hoon DSB, Wang Y, Dale PS, Conrad AJ, Schmid P, Garrison D, Kuo C, Foshag LJ, Nizze AJ and Morton DL (1995) Detection of occult melanoma cells in blood with a multiple-marker polymerase chain reaction assay. *J Clin Oncol* **13**: 2109–2116

Jung FA, Buzaid AC, Ross MI, Woods KV, Lee JJ, Albitar M and Grimm EA (1997) Evaluation of tyrosinase mRNA as a tumour marker in the blood of melanoma patients. *J Clin Oncol* **15**: 2826–2831

Leibovitz A, Stinson JC, McCombs (III) WB, McCoy CE, Mazar KC and Mabry ND (1976) Classification of human colorectal adenocarcinoma cell lines. *Cancer Res* **36**: 4562–4569

Mellado B, Colomer D, Castel T, Munoz M, Carballo E, Galan M, Mascaro’ JM, Vives-Corrons JL1, Grau JJ and Estape’ J (1996) Detection of circulating neoplastic cells by reverse-transcriptase polymerase chain reaction in malignant melanoma: association with clinical stage and prognosis. *J Clin Oncol* **14**: 2091–2097

Palmieri G, Strazzullo M, Ascierto PA, Satriano SMR, Daponte A and Castello G (1999) Polymerase chain reaction-based detection of circulating melanoma cells as an effective marker of tumour progression. *J Clin Oncol* **17** 304–31

Rankin EM (1996) Detection of micrometastases in malignant melanoma. *Eur J Cancer* **32A**: 1627–1629

Reinhold U, Ludtke-Handjery H-C, Schnautz S, Kreysel H-W and Abken H (1997) The analysis of tyrosinase-specific mRNA in blood samples of melanoma patients by RT-PCR is not a useful test for metastatic tumour progression. *J Invest Dermatol* **108**: 166–169

Rogan AM, Hamilton TC, Young RC, Klecker RW and Ozols RF (1984) Reversal adriamycin resistance by verapamil in human ovarian cancer. *Science* **65**: 994–996

Smith B, Selby P, Southgate J, Pittman K, Bradley C and Blair GE (1991) Detection of melanoma cells in peripheral blood by means of reverse transcriptase and polymerase chain reaction. *Lancet* **338**: 1227–1229

Van der Velde-Zimmermann D, Roijers JFM, Bouwens-Rombouts A, De Weger RA, De GraafPW, Tilanus MGJ and Van den Tweel JG (1996) Molecular test for the detection of tumour cells in blood and sentinel nodes of melanoma patients. *Am J Pathol* **149**: 759–764

Wang X, Heller R, VanVoorhis N, Cruse CW, Glass F, Fenske N, Berman C, Leo-Messina J, Rappaport D, Wells K, DeConti R, Moscinski L, Stankard C, Puleo C and Reintgen D (1994) Detection of submicroscopic lymph node metastases with polymerase chain reaction in patients with malignant melanoma. *Ann Surg* **6**: 768–774

*© 2000 Cancer Research Campaign*
